# Trophic overlap between expanding and contracting fish predators in a range margin undergoing change

**DOI:** 10.1038/s41598-018-25745-6

**Published:** 2018-05-21

**Authors:** Mats Westerbom, Antti Lappalainen, Olli Mustonen, Alf Norkko

**Affiliations:** 1 Tvärminne Zoological Station, J. A. Palménintie 260, 10900 Hanko, Finland; 2 Natural Resources Institute Finland, Latokartanonkaari 9, 00790 Helsinki, Finland; 3 Baltic Sea Centre, Stockholm University, Stockholm, Sweden

## Abstract

Climate change is predicted to cause a freshening of the Baltic Sea, facilitating range expansions of freshwater species and contractions of marine. Resident marine flounders (*Platichthys flesus*) and expansive freshwater roach (*Rutilus rutilus*) are dominant consumers in the Baltic Sea sublittoral where they occur in partial sympatry. By comparing patterns of resource use by flounders and roach along a declining resource gradient of blue mussels (*Mytilus trossulus*) our aim was to explore predator functional responses and the degree of trophic overlap. Understanding the nature of density-dependent prey acquisition has important implications for predicting population dynamics of both predators and their shared prey. Results showed a highly specialized diet for both species, high reliance on blue mussels throughout the range, similar prey size preference and high trophic overlap. Highest overlap occurred where blue mussels were abundant but overlap was also high where they were scarce. Our results highlight the importance of a single food item - the blue mussel - for both species, likely promoting high population size and range expansion of roach. Findings also suggest that range expansion of roach may have a top-down structuring force on mussels that differ in severity and location from that originating from resident flounders.

## Introduction

Investigating the causes and consequences of shifts in species range boundaries is a key ecological undertaking in a world facing increasingly large-scale ecosystem change. Many species have broadened their range due to anthropogenic impact while the range of others have contracted^1,2^. Given that range expansions and contractions are increasing^3–5^, understanding how species respond to impending ecosystem change has become increasingly important^6^. Range expansions potentially have large ecosystem effects^4^. They may proceed slower than invasions but their community level effect may be as far-reaching^4,7^. Especially shifts involving predators can be large and surprising since changes in predator species composition and abundance may affect an array of important ecosystem services and functions, even resulting in the loss of such services^8,9^. The ecosystem effects of wolfs returning to their natal habitats in Yellowstone caused a dramatic recovery of riparian plant communities with positive effects on biodiversity, even affecting stream morphology^10^. Re-introduction of wolves also affected grizzly bears positively through higher availability of plant-based food, initially caused by a wolf induced decline in the elk population that competed for those resources with bears^11^. A classic example from the marine realm was presented by Estes and colleges^12^ showing that the decline in the otter population caused sea urchins to increase, which through intense grazing deforested kelp beds. Sea otters for their part were reduced by increased killer whale predation. These cases illustrate well the complex linkages within ecosystems and the diverse function predators play in these systems. Shifts in predators likely cause shifts in ecosystem functions as some are uniquely linked to the presence of specific predators^13^.

As in many other aquatic ecosystems, fish play an important role in the Baltic Sea where they serve as a link between several trophic levels. As shown by Eriksson *et al*.^14^, by overfishing large piscivorous fish in the Baltic Sea, small bodied fish have increased considerably which has reduced grazers and their top-down impact on ephemeral algae causing nuisance algae to increase substantially in biomass^14^. Just as on terrestrial land^10,11^, only recently have we in marine systems begun to realize the complex role predators have on ecosystem function, ecosystem resilience and ecosystem health^14,15^.

Predicting how climate change will affect the range of species is a great challenge in all ecosystems. A key to determine the trophic outcome of range shifts is to unravel the effects of resource availability on species feeding behaviour and to elucidate how similarity in resource use affects the interaction between expansive and resident species. In peripheral regional seas, such as the Baltic Sea, the ecosystem is characterized by a few functionally dominant species that are living at the edge of their environmental tolerance limits to both salinity and temperature^16^. As the biota is a mixture of marine, brackish and freshwater species with species-specific tolerance limits to salinity, salinity changes may strongly affect species distribution patterns^16^. Given the severe conditions prevailing for both marine and freshwater species in this ecosystem, the Baltic Sea offers an ideal environment for the study of range dynamics and ecosystem change.

In this study, we examine the diet of flounder (*Platichthys flesus*) and roach (*Rutilus rutilus*), two partially co-occurring fish species in the shallow Baltic Sea sublittoral. Flounders and roach have previously been characterised by disparate distribution. Currently, they coexist in large stretches of the northern Baltic Sea. Over the past 30 years, freshwater roach has become increasingly abundant in coastal waters and a conservation concern because of its opportunistic behaviour showing high plasticity^17,18^. Monitoring data since the 1990s shows that the abundance of roach in outer coastal waters has increased by more than an order of magnitude whereas numbers of flounders have concomitantly declined dramatically^17,19^ (see Supplementary Fig. S1, unpublished national monitoring data by the Natural Resources Institute). This change has taken place with a parallel decline in sea surface salinity. Alongside with a geographical shift, there has also been a regional range expansion of roach and other cyprinid species towards more open outer archipelago areas that previously were dominated by marine fish –such as flounders^20^. The reason for this shift in dominance is believed to be eutrophication, but also ongoing changes in climate favour roach and other cyprinids that benefit from higher water temperatures and declining salinity caused by a change in climate^18,21^. Flounders and roach in the outer archipelago areas of the Baltic Sea feed on blue mussels^20,22^. Where their ranges overlap there is potential for significant similarity in resource use, such as marine blue mussels that also show declines driven by declining seawater salinity^23^. A high similarity between coexisting species may lead to competition and resource partitioning especially when commonly used resources become sparse^24^.

Despite the abundance of both species in this ecosystem, little is known about the feeding behaviour of adult flounders and roach where their ranges overlap. Importantly, no previous studies have actually quantified the potential overlap in distribution and feeding. Considering the expanding roach population towards the open archipelago area^20^, we predict to find similarity in resource use between the species and potentially overlap in diet. Using stomach content analyses, our study was designed to determine (1) the diet and prey-size selection of flounders and roach when feeding on a variable supply of blue mussels and (2) to estimate the degree of food overlap between sympatric flounders and freshwater roach. Specifically, we were interested in (3) relating the abundance of blue mussels with the foraging behaviour of both predators. Our hypothesis was that there is an intraspecific diet variation between areas with different availability of blue mussels. As an omnivorous feeder on detritus, plant matter, plankton and benthic animals^25^, we expected to find a more versatile diet among roach and less plasticity in flounder diet. In line with general theory and empirical evidence, we predicted that the overall niche width of both species would expand towards areas of lower mussel availability, but the expansion would be larger for roach. Ecological and behavioural studies on feeding behaviour between and among predator species have generally been done by studying either prey size (morphology) or prey diversity (composition). Because ecosystem change involves both aspects, we included both variables into this study. By comparing two consumers sharing a common resource across environments with different availability of this resource, we were able to evaluate the effects of blue mussels on fish feeding behaviour. Our aim with this paper is to increase the knowledge of the effects of ecosystem change on food web ecology in range margins, a theme that currently impedes our ecological understanding of natural ecosystems^7,16,26–29^.

## Materials and Methods

### Study system

**Figure 1:**
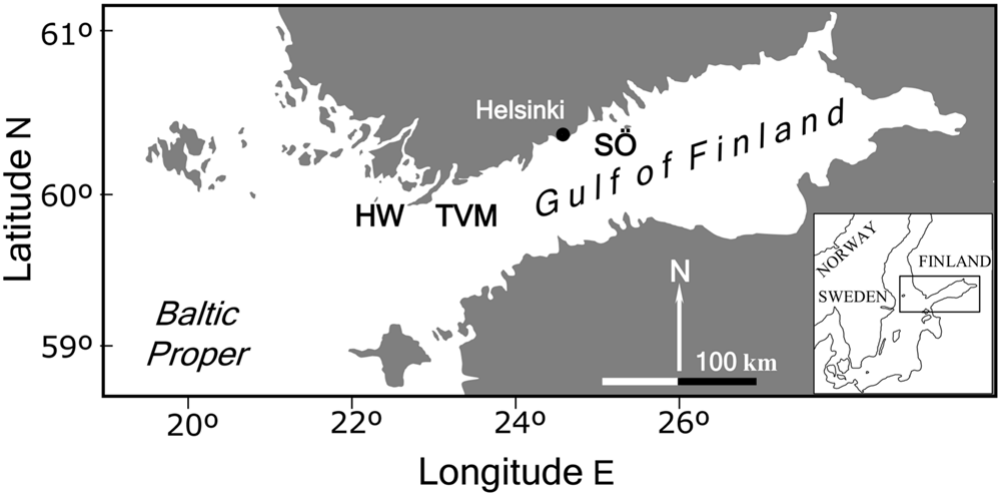
Map of the area. The study was carried out at Hanko Western (HW), Tvärminne (TVM) and Söderskär (SÖ) in the outer archipelago area of south Finland.

The study was carried out in the central and western Gulf of Finland, northern Baltic Sea. The brackish Gulf of Finland is characterized by a natural gradient in salinity. This gradient forms a clear transition zone between brackish-marine biota in the west and brackish-freshwater biota in the central and eastern parts of the Gulf. Our focal research area was situated at Hanko Western (59°55′N, 22°50′E, henceforth HW) at the entrance to the Gulf of Finland, Tvärminne (59°55′N, 23°15′E, henceforth TVM) and Söderskär in the central Gulf of Finland (60°07′N, 25°25′E, henceforth SÖ) (Fig. 1). Different abundance and size structure of blue mussels characterise the three areas with decreasing biomass towards the east and a sharp decrease in the abundance of larger mussels when moving from HW to TVM^23^. There is also persistently larger mussels at HW compared to TVM, advocating the inclusion of the two adjacent areas. These changes are driven mainly by salinity that varies from 5 ppt or less at SÖ to around 6 ppt at TVM and HW. Blue mussels are by far the biomass dominant macrofaunal species on these rocky bottoms throughout the gradient forming 90–95% of the animal biomass in the west^30^.

The significant increase in the dominance of roach in the outer archipelago has been demonstrated in previous studies. To provide a snapshot along the marine to the more limnic conditions in the core area and to show the relative dominance shift of roach relative to flounders in the area, fishing data from the national monitoring programme and university survey programmes were accessed. This monitoring data include 4 monitoring areas (each including 30–45 CPUE per annum) sampled with Nordic Coastal Multimesh Gillnets during the years 2005–2007. Offshore monitoring data on sea surface salinity were obtained from the Finnish Environment Institute.

### Quantifying fish diet

Fish sampling for diet analyses were carried out in May–November 2000. Due to unequal catches of larger fish between areas, sampling was continued in 2001 at HW and SÖ. Since initial catches were extremely low at depths exceeding approximately 10 metres, overnight fishing was carried out at shallow depths (3–10 m) close to rocky offshore islands. Flounders show high site fidelity, usually moving <100–200 m within a day^31^. Also roach was expected to be gregarious around the islands and not to migrate any significant distances over night as roach mainly is a day active species^25^. The low catches at further distances from the shores also indicated restricted mobility. Stomach content analysis is regarded as the most reliable method to determine fish feeding, especially in estuarine conditions^32^.

Within each area (i.e. HW, TVM and SÖ, respectively), we sampled three sites dispersed by a distance of 2–3 km. Fishing was then carried out from dusk until dawn at 2–5 locations within sites. For sampling, we mainly used Coastal survey nets (mesh sizes 17–50 mm from knot to knot) but also ordinary gill-nets with larger maximum mesh size (mesh sizes 25–60 mm) were used in order to specifically catch large specimens that were few in multi mesh gill-net catches. To terminate the activity of the alimentary canal caught fish were euthanized and put on ice. In laboratory, fish were measured for length and weight. Digestive tracts were removed and stored in 70% ethanol for later diet analyses. For flounders, we used the gut, whereas for roach only the anterior third of the intestine was used as roach lack a distinctive stomach. Gut contents were emptied into a Petri dish and identified to the lowest taxonomic level possible under a dissecting microscope. In flounders, volume proportions were estimated visually and individual counts were performed. Since roach masticate its food, estimating absolute numbers of food items is impossible. Therefore, only volume proportions were used in diet analyses when diet similarities between species were compared. Since season may affect the diet of fish, we also sampled fish at TVM in early May and late November in order to validate the summer diet (June–August). In all analyses, only fish with stomach contents were included. We therefore omitted 32%, 11% and 21% of flounders and 22%, 31% and 40% of roach at HW, TVM and SÖ, respectively.

### Blue mussel availability

Using SCUBA, we measured the availability and abundances of blue mussels by taking 12 random bottom samples from each of 3 sites in each area where fish sampling had been conducted. Samples were taken in July 2000 at 6–8 metres depth from rocky sublittoral shores using a 20 ∗ 20 cm quadrat sampler. This depth interval represents the abundance and biomass peak of blue mussels in the study area^23^. Samples were then sorted, individuals were counted and measured for length according to Westerbom *et al*.^23^.

### Size selection of blue mussels

In addition to diet sampling, fish with intact shells or fragments of shells in the deeper parts of the intestine were used to assess size selection of blue mussels. The study of size selection therefore include a substantial additional dataset of fish not used in the part of the study focussing on diet composition. In samples from flounders, almost 70% of mussel shells were intact and could be measured directly (>4000 shells were measured). The remaining >30% (>2000 mussels) had to be estimated by measuring the thickness of the umbo, which later was used as a proxy for mussel length. As roach masticate their food, the size of consumed prey in roach were estimated based on umbo thickness. A regression between known mussel size and umbo thickness was initially established by grinding umbos with manicure files and when reaching the thickest part this was measured using an ocular measuring scale (20 μm divisions) of a binocular microscope. The strong regression (r^2^ = 0.94, n = 230 for TVM and r^2^ = 0.91 for SÖ, n = 230) was later used to estimate the original length of blue mussels. Due to the proximity between HW and TVM, the same regression was used for HW and TVM. Since the number of intact mussel shells, or umbos gut^−1^ varied highly (1–230), we used average sizes gut^−1^ for statistical comparisons.

To analyse the preference of different fish sizes for different mussel classes we calculated Chesson’s electivity index (α) according to the formula:

1 $${\rm{\alpha }}=({r}_{i}/{n}_{i})/[\Sigma ({r}_{j}/{n}_{j})]$$

where *r* is the proportion of consumed mussels belonging to a particular size class and *n* is the proportion of that size class in the natural population. The index varies between 0 and 1 and weighs the preference of one mussel size category to the average preference for the alternative size category^33^.

### Diet composition and overlap

Interspecific diet overlap between flounders and roach was examined using several approaches. In all comparisons focussing on potential food overlap, we used the volume percentage composition of both flounders and roach. First, Schoener’s index (SI) was used to evaluate the magnitude of intra- and interspecific food niche overlap in the study areas:

2 $$SI=1-0.5\times \sum _{i=0}^{n}|{p}_{xi}-{p}_{yi}|\,i=1,2\,\ldots .n$$

In the index: *p*_*xi*_ = the proportion (varying between 0 and 1) of food category *i* in the diet of species *x*, *p*_*yi*_ = the proportion of food category *i* in the diet of species *y*, and n = the number food categories. According to Wallace^34^, overlap values exceeding 0.6 should be considered biologically significant indicating similar diet.

Secondly, niche breadth was calculated with the standardised Levins index to evaluate the width of resource use between species and areas:

3 $$B=\frac{1}{{\sum }_{i=1}^{n}{p}_{i}^{2}}i=1,2\ldots \mathrm{..},n\,{B}_{A}=\frac{B-1}{n-1}$$

where B = niche breadth, *p*_*i*_ = the proportion of food category *i* in the diet, and n = the number of food categories. B_A_ is Levin’s standardised niche breadth and varies from 0 to 1. The value is closer to 0 when most of the prey items belong to the same species (specialised diet), and closer to 1 when the predator doesn’t discriminate among resources (generalist).

Finally, to provide an additional line of evidence to the results given by the two indexes, we used resemblance-based methods to analyse multivariate differences and similarities in diet composition between and among species and areas. To test for seasonal differences within species, we computed a multivariate analysis of similarities (ANOSIM). Based on rank similarities, ANOSIM examines how differences within months compare to differences between months. The resulting R value shows the importance of the difference between groups, being close to 1 when differences are large. While ANOSIM is advantageous in many cases, it does not test for interactions between factors and provides no information on whether factor effects on diet differences are independent or whether they are the result of a combination of several factors. Therefore, testing effects of area, sites within areas, species, and size of individuals, Bray–Curtis matrices were subjected to four-way crossed permutational multivariate analysis of variance (PERMANOVA) using type III sums of squares as the designs were unbalanced^35^. PERMANOVA calculates Pseudo-F from a distance/dissimilarity matrix and discriminates group differences. Where ANOSIM and PERMANOVA detected significant differences, similarity percentage analysis (SIMPER) was used to estimate the contribution of each prey taxa to any significant differences in the diet of each tested factor. An ordination diagram (PCO) was used to visualize the groupings from PERMANOVA as a measure of the distinctiveness among the groups in multivariate space^35^.

### Data analysis

Since initial tests showed no year effect in bottom samples^36^ neither within nor between species, year effects are not considered. Parametric tests were used in univariate analyses whenever test requirements were met. In most cases, we couldn’t meet the requirements for parametric tests. In these cases we used non-parametric one factor Kruskal-Wallis tests. As the diet was estimated as proportions, the non-parametric Scheirer-Ray-Hare test (henceforth S-R-H) was used in 2 or 3-factor analyses when testing for niche breadth and species richness. We used permutational analysis of variance (PERMANOVA) or analysis of similarities (ANOSIM) to test for similarities in composition between species and areas. Apart from the sites, which were treated as a random factor nested within area, factors were fixed and crossed. SIMPER was then used for examining similarities in diet between species and areas. All multivariate analyses (ANOSIM, PCO, PERMANOVA, SIMPER) were run in PRIMER v6. Prior to testing, data were square root transformed to account for variation in species abundances, subjected to the Bray-Curtis resemblance matrices, and checked for group homogeneity^35,37^.

Randomized cumulative prey curves were used to ascertain that the stomach numbers adequately represent the regional diet^38^. A cumulative prey curve reaches an asymptote when an increase in sample size doesn’t increase the power of interpretations. The order in which stomachs were analysed was randomized 999 times. The cumulative prey items curve for flounder and roach fitted better with logistic non-linear regressions (R^2^ = 0.97, 0.99, 0.99 respective R^2^ = 0.98, 0.96, 0.99) for the three areas HW, TVM, SÖ, than with linear regressions (R^2^ = 0.70, 0.76, 0.8 respective 0.67, 0.63, 0.68, p ≤ 0.001 in all cases). For both species and all areas, an asymptote was reached well before the total sample size, showing that sample size at all areas was sufficient to reliably describe the diet of both species. In all analyses, individual fish was the sampling unit used in figures and tests.

Data on roach diet has been reported previously in Lappalainen *et al*.^39^ and Westerbom *et al*.^36^, but here we considerably expand on previously published data with new data that facilitates a quantitative comparison of feeding biology between the two species. The datasets generated during the current study are available from the corresponding author on reasonable request. All necessary permits were obtained for the described field studies and work was carried out according to ethical principles and guidelines by LAC (Laboratory Animal Centre of the University of Helsinki). Specifically, the study was performed under the relevant guidelines and regulations from the National Animal Experiment Board of Finland and its licensing committee (licence to the first author).

## Results

### Spatial trends in fish population size

**Figure 2:**
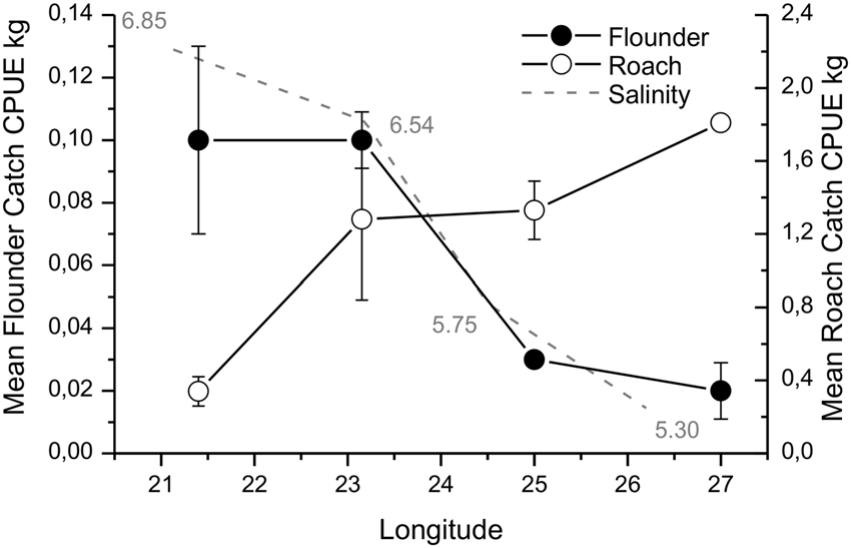
Population trends in flounder and roach (average ± SE CPUE) along the south coast of Finland during the years 2005–2007. Long term sea water salinity levels from open sea are indicated in grey. Note the difference in scale of both Y axes.

Along the salinity gradient, the abundance of flounders declined towards the east (Fig. 2), largely following ambient declining seawater salinity. Roach on the other hand showed an opposite pattern, increasing towards areas of lower salinity. Throughout the coastline roach outnumbered flounders manifold in terms of CPUE.

### Distribution of blue mussels

**Figure 3:**
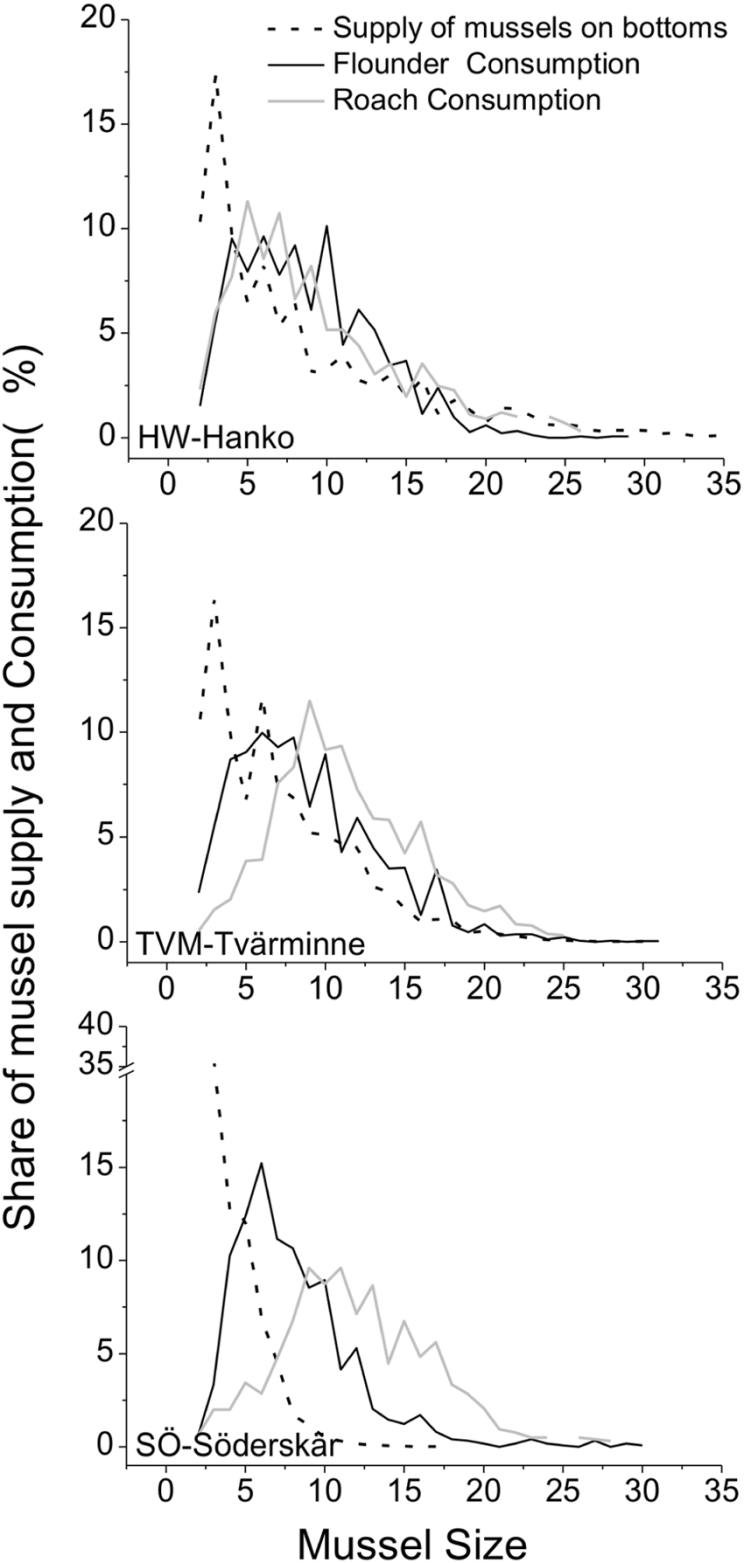
Population structure of blue mussels at the three areas and summed consumption from the two sympatric predators. No error bars are shown to ease separation of lines and interpretation. Mussel size in mm.

Densities of blue mussels differed between the areas (S-R-H; H_2_ = 43.7, p ≤ 0.001) with highest occurrence at TVM and lowest at SÖ (14.6 k ± 1.0, 22.2 k ± 2.1 and 9.1 k ± 0.7 k at HW, TVM and SÖ, respectively). Because of a much more even size distribution in the west (Fig. 3), including also large individuals, biomasses declined from west to east (ANOVA, F_2,105_ = 70, p ≤ 0.001) being 73 ± 31 gm^−2^, 61 ± 37 gm^−2^ and only 5 ± 3 gm^−2^ at the respective areas HW, TVM and SÖ. The difference in size distribution between the areas was clear. At HW, 17% of the mussels exceeded 15 mm in length, at TVM only 5% and at SÖ less than 1 per mille were larger than 15 mm. Conversely, mussels smaller than 10 mm contributed with 64%, 69% and 99% to the population size at the respective areas HW, TVM and SÖ.

### Diet of the two predator species

Diet analysis for a total of 316 flounders and 357 roach showed similar food types across areas and species. Both fish fed on the same resources in all areas, but the resources appeared in slightly different proportions depending on species and area. Shelled molluscs, especially blue mussels, clearly dominated the diet of flounders in all areas. Over 80% of flounders at HW, 90% at TVM and more than 60% at SÖ consumed blue mussels (see Supplementary Table S1). On average, 6.1 ± 8.9 (SD), 15.2 ± 16.7 and 5.9 ± 11.0 individuals of blue mussels were found in the guts of flounders at the respective areas HW, TVM and SÖ. Of other species, only *Macoma balthica* was commonly used by flounders in all areas, with an average of 3.3 ± 8.2 (SD), 4.0 ± 8.7 and 2.9 ± 8.3 individuals in the respective areas HW, TVM and SÖ. Isopods (particularly *Saduria entomon*) but also amphipods (Gammarids) were abundant in some individuals from the eastern area, whereas the role of these species was less pronounced in the two western areas.

Roach was also clearly molluscivorous, with blue mussels forming the single most important food item, being found in 66%, 91% and 76% of the roach at the respective areas. The occurrence of gastropods also had a significant share in roach diet (see Supplementary Table S1).

### Community effects

**Figure 4:**
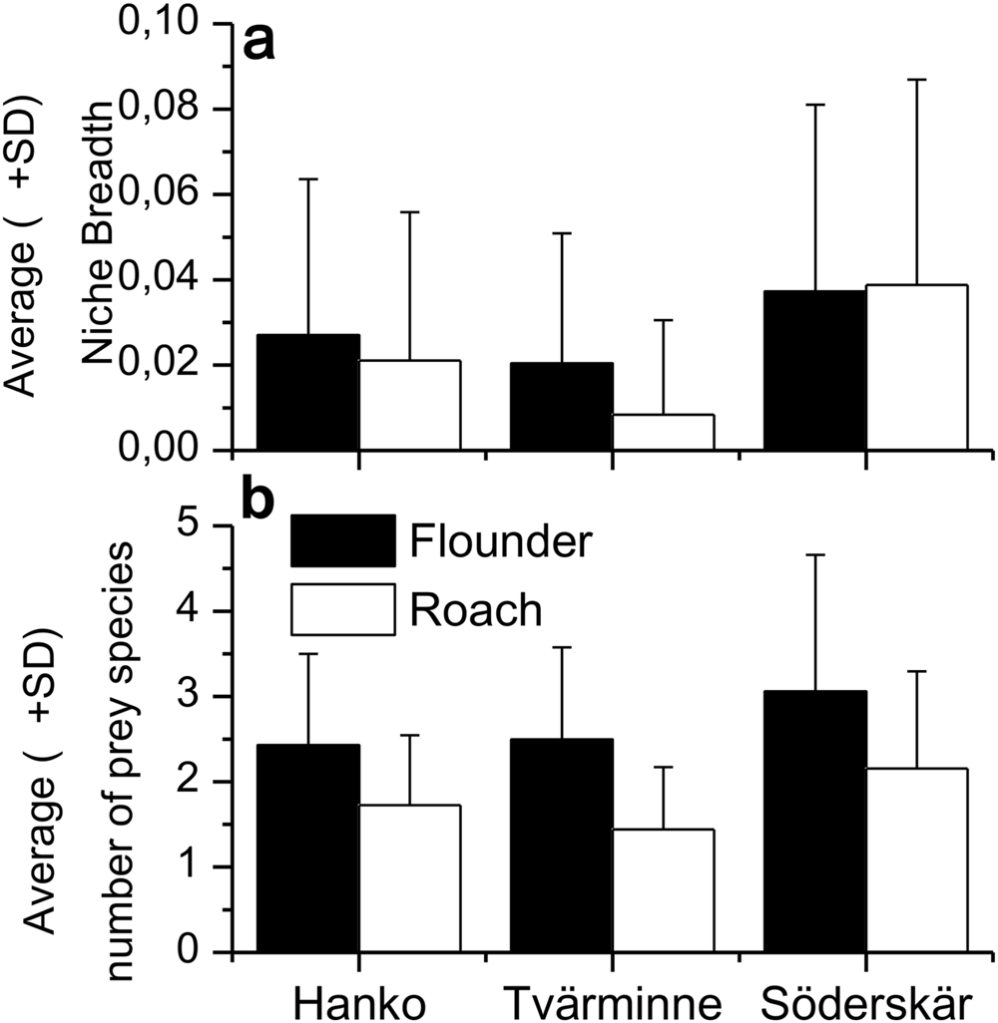
Niche breadth (**a**) and species richness (**b**) in diet of flounders and roach in the three areas.

The species had diet overlaps (Schoener’s index) of 44% at HW, 77% at TVM and 61% at SÖ, indicating a significant overlap in trophic niche at TVM and SÖ. Niche breadth (Fig. 4a) was very small for both species, mirroring the dominance of blue mussels in the diet throughout the range. Niche breadth differed between species (S-R-H test: H_1,656_ = 24.0, p ≤ 0.001) and across areas (H_2,656_ = 56.0, p ≤ 0.01) but showed no interaction between area and species and no effect of fish size. Both species consumed more species at SÖ compared to both HW and TVM (H_2,656_ = 13.2, p ≤ 0.01, Fig. 4b) and flounders consumed more species than roach (H_1,656_ = 56.0, p ≤ 0.001) with no interaction between species and area (H_2,656_ = 0.84, p = 0.65).

Four factor PERMANOVA showed significant differences in diet between species and areas (Pseudo-F_1,629_ = 34.9, p = 0.003, respectively Pseudo-F_2,629_ = 6.9, p = 0.02). Additionally there was a significant interaction between species and area (Pseudo-F_2,629_ = 14.0, p = 0.002). Pairwise tests showed that the diet between the species differed at HW (p = 0.01) and SÖ (p = 0.001), but showed no difference at TVM (p = 0.26). They also showed that the diet of roach differed between HW and TVM (p = 0.039) and between HW and SÖ (p = 0.001) but not between TVM and SÖ (p = 0.112). The diet of flounders were non-significant in all pairwise comparisons between areas (HW and TVM p = 0.643, HW and SÖ p = 0.107 and TVM and SÖ p = 0.104). PERMANOVA also showed no effects of fish size (3 categories) on overall diet in either of the species (Pseudo-F_2,629_ = 1.3, p = 0.289). Also diets among sites within areas, were homogenous (Pseudo-F_6,629_ = 1.3, p = 0.199).

**Figure 5:**
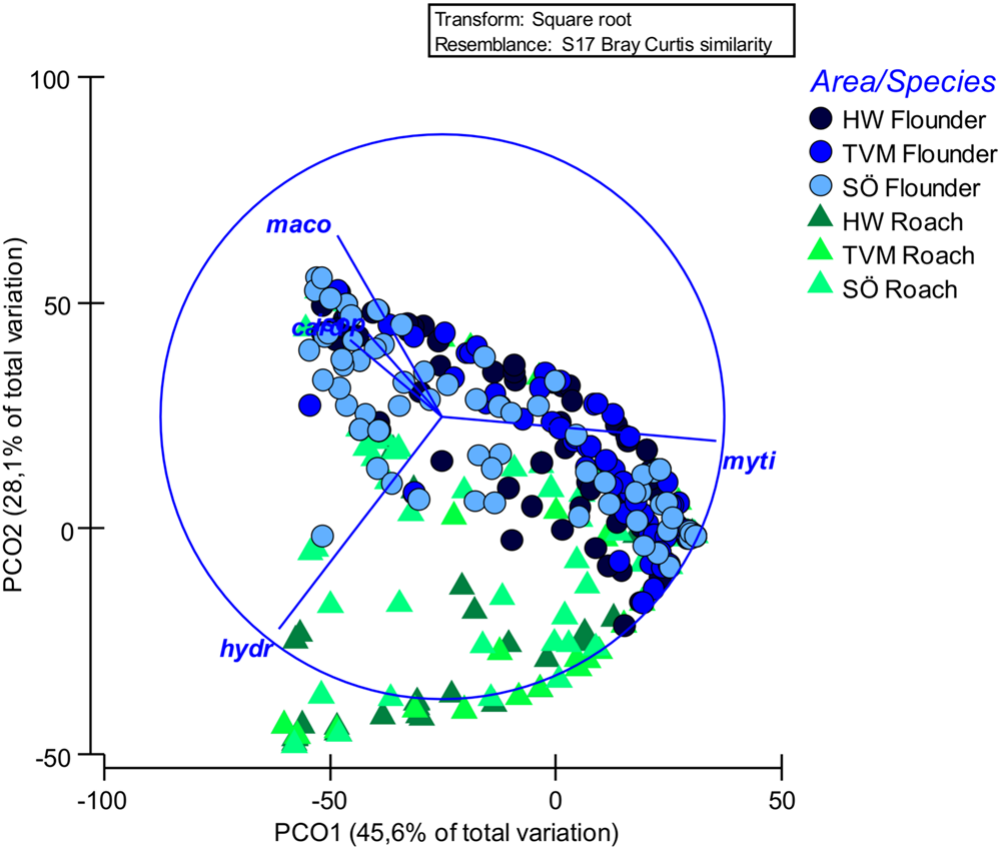
Principal Coordinate Ordination showing location and dispersion of the diet composition in fish and area. PCO3 (z-axis) explained 12% of the total variance (totalling 86%). Card = Cerastoderma, hydr = Hydrobia, iso = Isopoda, maco = Macoma, myti = Mytilus.

The similarity in diet was also seen in the PCO ordination (Fig. 5) where flounders and roach were grouped in one cluster. Small differences in diet were shown by the PCO as the two species were basically grouped at different ends of the cluster. The PCO ordination revealed that the variance among flounders was mainly due to differences in the use of *M*. *trossulus* and *M*. *balthica*, whereas for roach variance was mainly explained by how individuals used *M*. *trossulus* and *Hydrobia* sp. At SÖ, *Cerastoderma glaucum* and *Saduria entomon* caused some significant variation among flounders.

Analysing within-area similarities, SIMPER demonstrated that the diversity of ingested prey species was highest at the easternmost (SÖ) area (35% for roach and 32% for flounders) for both predatory species where blue mussels are less dominant. In contrast, at TVM where blue mussels were most abundant, average similarities were highest (63% for roach and 54% for flounders) with blue mussels contributing most to intraspecific similarity. HW fell in between both TVM and SÖ (50% for roach and 46% for flounders).

Looking specifically on the feeding behaviour in relation to blue mussels; fish size didn’t affect the proportion of blue mussels in the stomach of flounders at HW and TVM. However it was negatively correlated at SÖ (Spearman rs = −0.32, n = 80, p = 0.003) indicating that the lower availability of preferable sized blue mussels for larger flounders at SÖ are compensated by other prey. Indications of similar effect was found for roach but this was not significant (Spearman rs = −0.2, n = 71, p = 0.06).

### Seasonal effects

There were significant, but very small differences in diet between seasons. ANOSIM showed that summer diet in flounders, June–August, differed from both May and November (Global R = 0.18, p = 0.08%) with negligibly small R-values in pairwise comparisons, indicating very strong overlap between seasons. No differences were seen between diets within the season June to August (p = 0.33) and neither between the cold-water periods May and November (p = 0.63) but a slight difference was found between the warm water periods June and August compared to November (R = 0.23, p ≤ 0.01, respectively R = 0.38, p ≤ 0.01). Roach showed small seasonal effects (ANOSIM, Global R = 0.09, p ≤ 0.001), indicating some but very small inter-annual differences in diet. The diet in roach was similar in June – August (p = 0.23) and equally to flounders showed higher R-values in pairwise comparisons between June and August compared to November (R = 0.23, p ≤ 0.001, respectively R = 0.23, p ≤ 0.001).

There was a strong overlap in species richness of selected prey by flounders between seasons (S-R-H test: H_3,136_ = 3.3, p = 0.34). The volume proportion of *M*. *trossulus* and *M*. *balthica* however changed during the season. In November, flounders decreased the consumption of *M*. *trossulus* compared to August (Kruskal-Wallis H_3_ = 13.98, p ≤ 0.01) whereas they decreased the consumption of *M*. *balthica* from May to August (Kruskal-Wallis H_3_ = 8.34, p = 0.04). Roach showed small seasonal effects, indicating some inter-annual differences in diet composition (S-R-H test: H_3,253_ = 12.7, p = 0.005) with November differing from August. The volume proportion of *M*. *trossulus* dropped markedly in November (from >80% in June to 28% in November, Kruskal-Wallis H_3,253_ = 48.4, p ≤ 0.001), whereas the predation on Isopods (0% to 26%, Kruskal-Wallis H_3_ = 67.6, p ≤ 0.001) and Hydrobids (6% to 34%, Kruskal-Wallis H_3_ = 14.4, p ≤ 0.01) increased. *M*. *balthica* was consumed much more in May and June than in November (19% to 0.1%) (Kruskal-Wallis, H_3_ = 22.3, p ≤ 0.001).

### Prey-size selection

**Figure 6:**
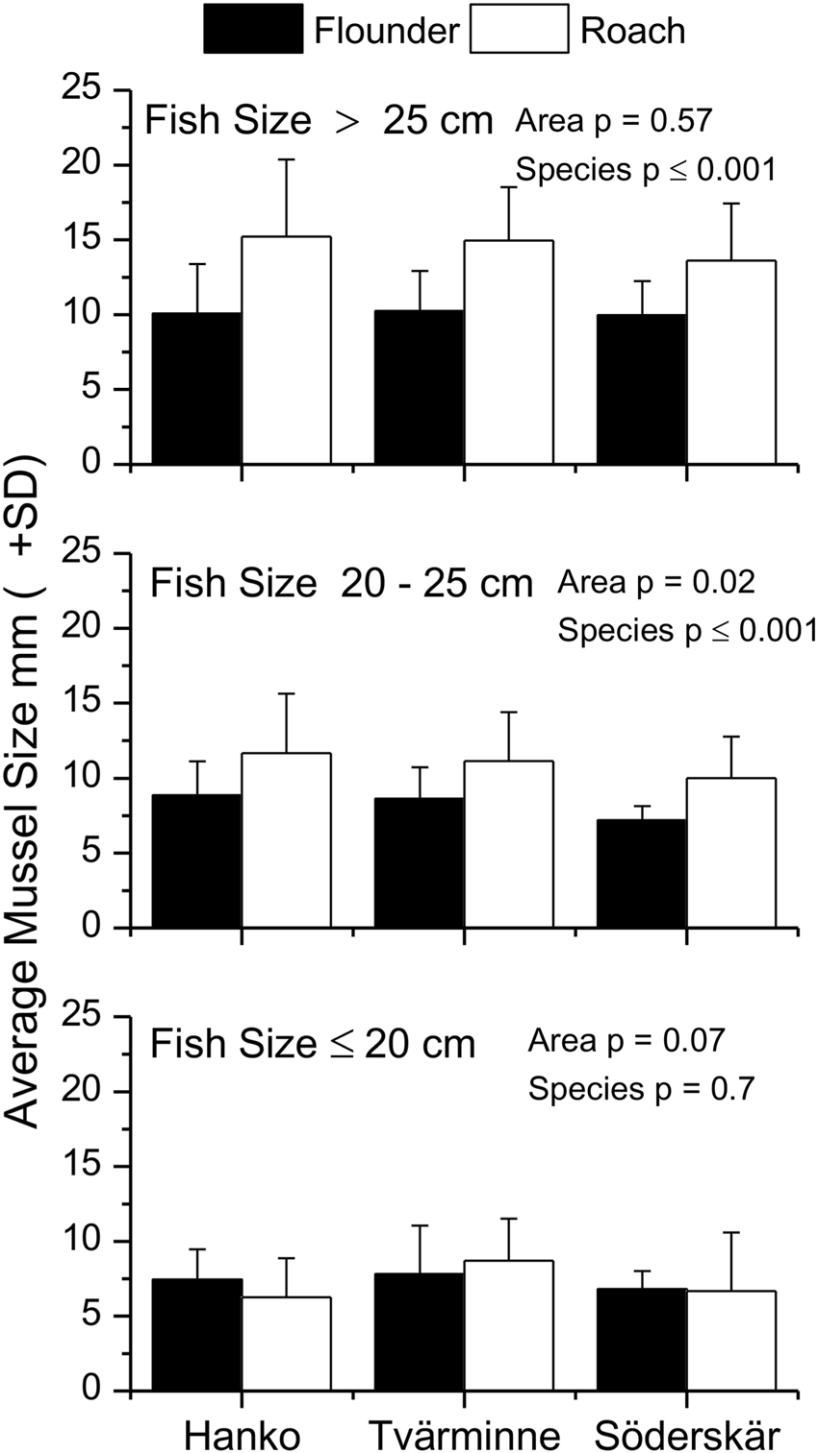
Effect of fish size on the size of consumed blue mussels at the three areas.

To study the effects of area, species and fish size on prey-size selection, the data were grouped into three classes of fish (small: less or equal to 20 cm, medium >20–25 cm and large >25 cm). The size of ingested blue mussels ranged from 2 to 30 mm for both species covering the size distribution of blue mussels in the area. The size of ingested prey was strongly affected by fish size (S-R-H test H_2,621_ = 127.6 p ≤ 0.001) and species (S-R-H test H_1,621_ = 43.95 p ≤ 0.001, Fig. 6), but no interactions were shown. Roach consumed larger mussels than flounders and area differences were significant (S-R-H test H_2,621_ = 7.75 p = 0.02) because fish at SÖ ate smaller mussels compared to those at TVM and HW.

**Figure 7:**
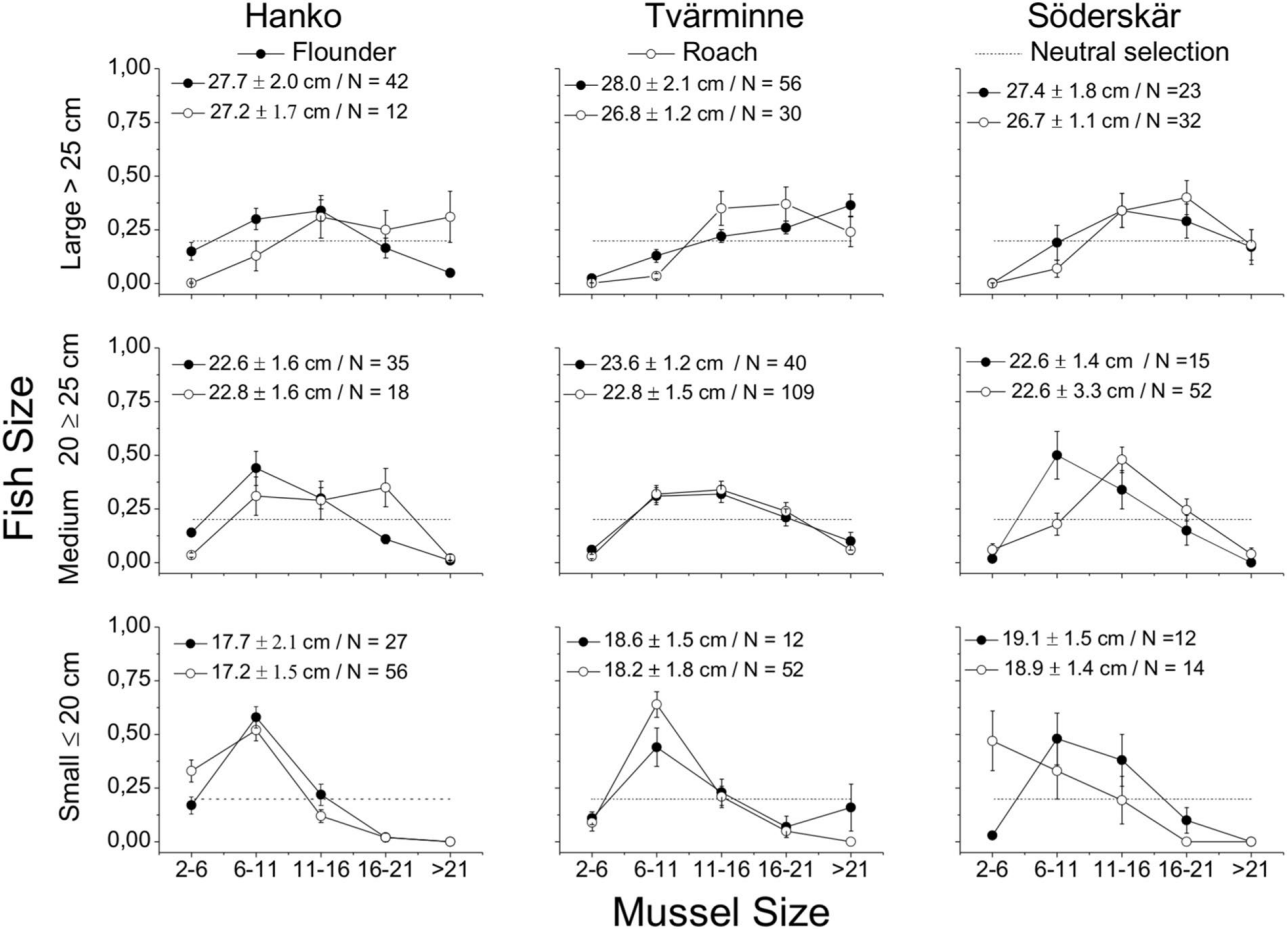
Chesson’s index of preference for different length classes of mussels selected by different length classes of flounders and roach (SL) at the three areas. Neutral selection (dashed line) is defined as 1/number of size classes (5). Alfa values above the line indicate selection for a size class and those below it indicate selection against. Mussel size in mm.

Chesson’s index of preference (Fig. 7) indicated that (1) flounders and roach generally were selective towards the medium sized mussels, (2) preferences towards larger mussels increased with the size of the predator and (3) the preference towards larger mussels increased slightly from west to east. Chesson’s index further showed that the preferences between flounders and roach were largely similar although roach tended to prefer on average slightly larger mussels. The index also showed an increasing preference for larger mussels with increasing fish size for both species and a general preference for mussels in the size range 6–21 mm. Schoener’s index computed on the population level, indicated strong interspecific overlap in the size of consumed mussels at HW (SI = 78%) and TVM (SI = 69%) but no considerable overlap at SÖ (SI = 42%).

## Discussion

Climate change is affecting the Baltic Sea, with increasing temperature, reduced sea ice cover in winter and a gradual freshening of the water. This change affects the distribution of species, especially those that live at the edge of their distribution margin^16^. We found that: 1) the distribution of the focal predatory species within the area reflected changes in ambient sea-water salinity and responses were disparate in shape (Fig. 2). Salinity with other concurrent changes such as temperature and eutrophication have resulted in a temporal decrease of flounders^19^ and increase of roach^18^. We further showed that: 2) blue mussels constituted a substantial portion of the diet in both predatory species along the studied gradient even in the eastern area where larger mussels were very few. We also found that: 3) there was a large overlap in resource use between both predatory species along the entire gradient, overlap being highest where mussel biomass peaked and where it was low. Finally, 4) the niche breadth of both species was very narrow throughout the resource gradient, indicating a specialized diet composed of only a few species in each individual fish. Individuals are specialists if they mainly feed on a few resources. They are regarded generalists when they utilize a wide variety of resources^33^. A clear and distinct response over the resource gradient was not seen in either of species, even if niche breadth was higher in the east where blue mussels showed lower occurrence. In the east, the large variance in niche metrics indicates that while eating only a few species at a time, the composition of the few species vary between individual fish, possibly indicating also disparate individual responses to mussel availability.

Environmental change, including changes in climate, inflicts changes in species’ ranges indirectly by e.g. causing increases in the number of competitors or predators or by changing the resource base that population size depend on. Several studies on biotic responses to climate change predict species redistribution and displacement away from the centre of the species home range^3,40^. Given that these range expansions and contractions ultimately lead to a cascade of biotic interactions, e.g. shifts in the competitive balance between species, a logic first step is to examine how species currently use their resources over resource gradients and how they respond to a change in the availability of these resources. In the present study, population changes of the two coastal fish species were clear across space following changes in salinity (Fig. 2). Even if correlation doesn’t infer causality and other explanations may abound, we illustrate the consistent trends of increasing roach and simultaneously decreasing flounder population size with a concomitant decline in seawater salinity which holds true both temporally and spatially (see Supplementary Fig. S1, Fig. 2). The distribution of blue mussels and flounders are disadvantaged by low salinity conditions and both showed a marked decline towards low saline areas. In contrast, reproduction of freshwater roach is impaired by high salinity^18^ and population size declined towards the more saline areas (Fig. 2). Analogous changes were seen over time (see Supplementary Fig. S1). There is, therefore, an ongoing trend of increasing dominance of cyprinid species in the coastal ecosystem with a progressing freshening of the sea and a consequent weakening of the population size of flounders^18,19^. These changes will be amplified in the future Baltic Sea with predictions towards an increasingly warmer and limnic ecosystem^16,41^. Even if current environment-biota relationships don’t necessarily hold true under future climate change, the consequences of these population shifts possibly include the adverse effects of increased competition for declining resources and/or the possibility of expanding populations to use different resources from those whose range contract.

The study revealed that flounders in the shallow outer archipelago area show high reliance on blue mussels in all seasons almost throughout the distributional range of both predator and prey species. Borg *et al*.^22^ showed that the consumption of blue mussels in the shallow mosaic archipelago area, characterizing the outer Northern Baltic Sea, is not strictly limited to rocky shores, but blue mussels dominate the diet of flounders also in shallow outer archipelago areas where other bottom types dominate. Adult flounders have largely been shown to be almost strict benthivores dependent on molluscs, polychaetes, amphipods and isopods^42–45^. For roach, however, the capacity to be an opportunistic generalist is well documented^25,46,47^. Despite the capacity to consume a large array of different food items across a wide range of habitats, we have demonstrated that roach almost solely feed on blue mussels in the outer archipelago. Here we showed that the niche breadth was extremely narrow all over the area and there was a high reliance on blue mussels in the diet also in areas where preferable sized blue mussels were in short supply. Our results suggest that where the range of blue mussels and roach overlap, roach show a high reliance on blue mussels. This switch is turned on also in areas of low biomass of blue mussels. We also showed that when the main resource was extremely abundant offering almost endless amounts of preferable sized mussels, roach almost solely consumed blue mussels. Roach included a slightly more versatile diet when this resource was low, a response that is in line with theory saying that the dietary diversity of consumers increases as food becomes limiting. However, the shift towards other prey was not strong. Even at the eastern area with low biomasses of blue mussels and with an unfavourable size structure, blue mussels still dominated roach diet. This demonstrates that roach seems to use the resource over all its geographical range where the range of the prey and the predator overlap. We therefore conclude that in the outer shore areas of the Baltic Sea, flounders and offshore roach seem to occupy a very similar trophic position.

Besides being able to switch between food items, roach also utilises a broad habitat niche and migrate between habitats when feeding resources become low in one end of its habitat niche. Because of this flexibility, population size of roach stays high also during periods of lower availability of mussels – giving roach a competitive advantage over other molluscivores, whose population size is more controlled by benthic prey and whose regional ranges are much narrower. Flounders, for example, are less flexible in habitat and food choice and are constrained in distribution to the outer archipelago area.

It is known that high overlap in resource use between sympatric species increases the likelihood for exploitation competition when resources become limited^24,48^. As there is no conclusive way to ascertain the occurrence or magnitude of exploitative resource competition here or elsewhere^49^, such suggestion remains tentative. Nonetheless, the results of this study suggest that roach is exploiting resources that would otherwise be available to other molluscivores and it may therefore have a competitive effect at the margin of flounders where blue mussel resources are sparse. When looking at results in this study, the reduced availability of blue mussels along the resource gradient induced similar responses in terms of niche breadth for the two species, most likely caused by declining availability of blue mussels of preferable size (Figs 4a and 6). Both species broadened their diets at the eastern margin (SÖ) and showed the narrowest niche where the main food item was most abundant and of most preferable size (TVM). The occurrence of alternative prey differs to some extent between areas, the average species number at sites declining slightly in scuba samples from west to east (in line with Vuorinen *et al*.^16^), but also showing increases of some taxa, e.g. *Cerastoderma glaucum* in the east^50^. Despite small differences in composition, there are plenty of other species available on the seafloor throughout the range, indicating a general shift in resource use independent of the supply of alternative resources. Both species also increased the consumption of species in the east that show no differences between areas^50^.

Flounders exhibited a broader niche than roach at both western areas whereas roach exhibited a larger relative change in niche breadth from TVM to SÖ (Fig. 4a). At TVM, both species had diets based almost exclusively on blue mussels and they showed a very strong diet overlap. At the two other areas, diet overlaps were moderate or high, but lower than at TVM demonstrating higher diet segregation between the species in west and east compared to TVM. The reasons for the decline in the population size of flounders are uncertain and likely primarily caused by an overall freshening of the Baltic Sea causing reproductive failure^51–54^. Resource availability is not likely to explain the declining population, especially since declines have occurred along the entire range of flounders, also where mussels are in plenty. It is however evident that increased and escalating overlap in resource use may negatively affect the fitness of those molluscivores that show limited flexibility in diet. However, the magnitude of such effect remains unsolved since no reliable density measures of flounders nor roach exist for this or any other area in the Baltic Sea^55^.

Besides predator-predator interactions, predation is also a major structuring force. Functional response, i.e. how predation rate responds to changes in structure or abundance of their resources will apparently affect prey populations. Since predator-prey interactions are dependent on the physical and physiological environment within which they interact^9^, ecosystem changes may also alter community composition and the strength of species interactions. If ecosystem change permits predators to enlarge their range, there may be considerable effects on the prey populations they encounter, possibly suppressing the geographical range of some prey^7^. Invading omnivorous feeders may have an especially large impact because their fitness is not restricted to a few items but they can compensate declines in one resource base with other resources and therefore remain high population densities also in fluctuating conditions.

## Conclusions

**Figure 8:**
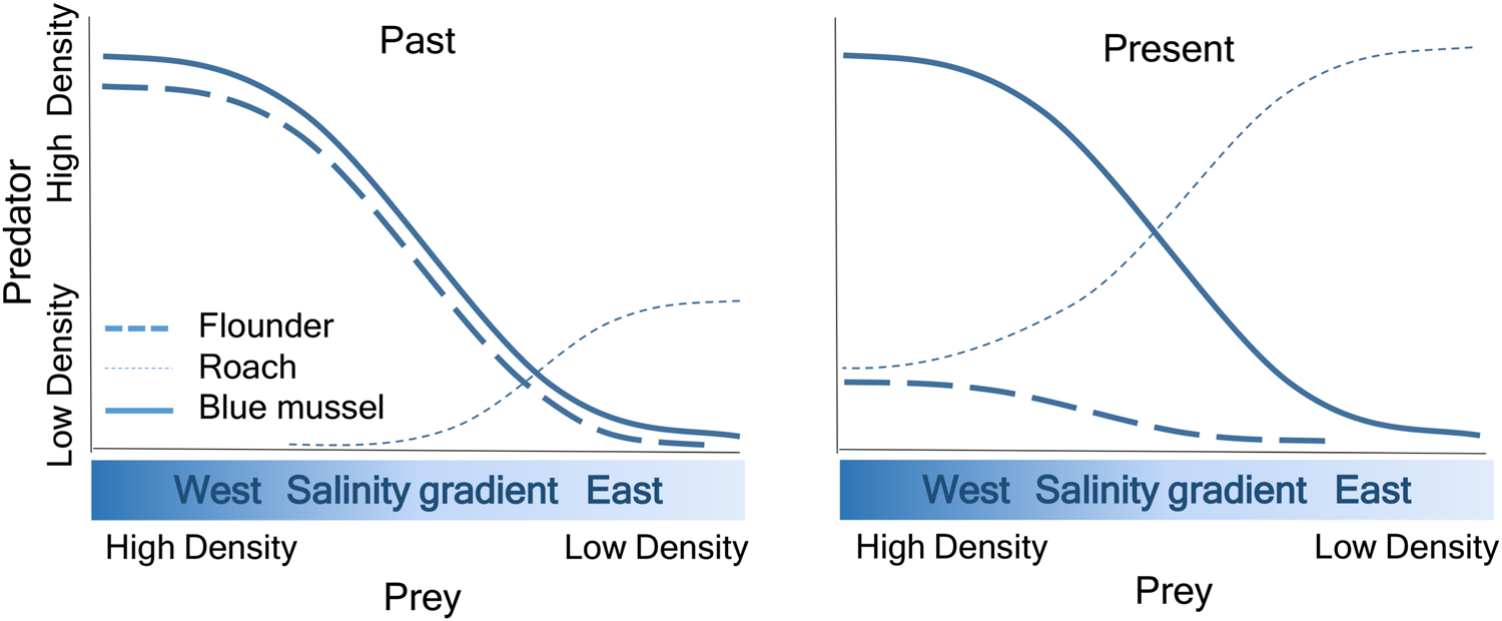
Conceptual model outlining the ecological interactions between prey, predator and predator impact. The past scenario indicates the situation when flounders were the only dominant fish predators on mussels in the outer archipelago and where predation pressure was equal along the range. The prey and the predator had a similar distribution pattern caused by salinity requirements and consequently the total predation pressure paralleled the distribution of both species. The present scenario describes the situation when a novel predator to the system or the region has a distribution pattern that contrasts the environmental requirement of its prey. In this case, it will exert increasingly higher predation pressure at the margin of distribution of its main prey, where prey is sparse and predators are highly abundant. In our case, in west, predation pressure is still low due to high biomasses of blue mussels. Predation pressure from flounders continues being weak because of a declining flounder population. If the resource base in future declines (the solid line drops), predation pressure from roach will increase and be increasingly significant towards the west. If also the population size of roach will increase, this will cause an even larger pressure on declining mussel populations.

Along the gradient in this study system, predation intensity is expected to be proportionately higher at the edge than at more central areas as the summed predator encounter rates become proportionately much higher towards the edge (Fig. 2). Predation on blue mussels has been considered unimportant in a Baltic Sea context. Ongoing ecosystem change may, however, severely modify predator-prey interactions. Previously, the study areas were mainly inhabited by flounders and other marine molluscivores whose geographic distribution and predation pressure largely paralleled that of blue mussels (Fig. 8). Such parallel synchrony in population size kept the predator impact in balance in relation to the resource. The ecosystem of today and tomorrow is however characterised by opportunist roach and other cyprinids whose predation pressure via higher population size increases as salinity declines, causing additional pressure on blue mussels on top of the intrinsic pressures linked to physiology and breeding success. Frequency-dependent predation; i.e. the switch to abundant prey types in response to temporal and spatial variations in resource availability, can maintain high populations of roach, even if blue mussels decline. In this scenario, roach will have an ever-increasing role in the Baltic Sea coastal ecosystem. Freshening of estuaries and coastal waters caused by climate change and the following shifts in species will result in different patterns of shifts compared to those driven by thermal changes. While climate-related shifts in fish distribution, through ocean warming, have been typically characterized by displacement away from the historical center of species (colonisation in leading edge and contraction of the trailing edge), species that benefit from freshening of the sea (like roach), will not *ceteris paribus* abandon their previous ranges but their home range will expand. Marine species, like flounders, on the other hand, will see a contraction of the trailing edge. Flounders will be displaced away from their historical home range caused by breeding problems in low salinity conditions, possible declining resource base, and perhaps increasing competition from roach and other cyprinids that compete for the same niches. Such shifts will also have socio-economic impacts, when a commercial species will decline and unharvested species will bloom.

## Electronic supplementary material

Supplementary Figure and Table
