# Supplementary material for: Trophic overlap between expanding and contracting fish predators in a range margin undergoing change
Source: Sci Rep. 2018 May 21;8:7895. doi: 10.1038/s41598-018-25745-6 (PMC5962582; doi:10.1038/s41598-018-25745-6)
Supplement: Supplementary file 1 — Supplementary Figure and Table [file 41598_2018_25745_MOESM1_ESM.docx]

**Supplementary material to:**

**Trophic overlap between expanding and contracting fish predators in a range margin undergoing change**

Mats Westerbom, Antti Lappalainen, Olli Mustonen, Alf Norkko

Correspondence to mats.westerbom@helsinki.fi

**Supplementary Figure S1**. Changes in fish populations over time from the outer Archipelago Sea. Different net-series were used in 1991 – 2004 and 2005 – 2016, with overlapping methodology in 2004. Trends, measured as CPUE, are therefore shown in two periods. Flounders showed a 3 fold decrease in abundance between 1991 – 2004 (rs = -0.72, p < 0.001) which continued even stronger in 2005 – 2016, (r = -0.81, p < 0.001). Roach showed a 16 fold increase between 1991 – 2004 (r = 0.87, p < 0.001) which levelled off but stayed high in 2005 – 2016 (r = -0.02, ns, trend lines in dash). Salinity measurement t-10 years show a decline over the entire period (r = -0.66, p < 0.001, trend line grey). Note the difference in scale in both Y axes. Methods are described in detail in Ådjers et al.^17^ (see reference in main article).

**Supplementary table S1.** Gut contents of flounders and roach expressed as percentage of guts containing a given food item. Differences between species within areas are tested with Chi2-test on frequencies for all variables with expected values > 5. For flounders, the average number of a given food item/gut is also given and tested with Kruskal-Wallis. * P < 0.05, ** P < 0.01, *** P < 0.001.
